# Evaluation of safety attitudes of hospitals and the effects of demographic factors on safety attitudes: a psychometric validation of the safety attitudes and safety climate questionnaire

**DOI:** 10.1186/s12913-019-4682-0

**Published:** 2019-11-14

**Authors:** Chuang Zhao, Qing Chang, Xi Zhang, Qijun Wu, Nan Wu, Jiao He, Yuhong Zhao

**Affiliations:** 1 China Medical University, Shenyang, China; 2 Shengjing Hospital of China Medical University, Shenyang, China; 3 Liaoning Province Medical Doctor Association, Shenyang, China; 4 The First Affiliated Hospital of China Medical University, Shenyang, China; 5 The First Affiliated Hospital of Dalian Medical University, Dalian, China

**Keywords:** Patient safety, Safety attitude questionnaire, Demographic factors, Psychometric properties

## Abstract

**Background:**

The objectives of this study are to test the psychometric properties of the safety attitudes and safety climate questionnaire Chinese simplified version (SAQ-CS), to test the safety attitudes of health professionals in tertiary hospitals in the Liaoning province and to explore the effects of demographic factors on safety attitudes.

**Methods:**

The SAQ-CS was used to conduct a cross-sectional survey in nine tertiary hospitals in Liaoning province.

**Results:**

Cronbach’s alpha of each subscale of SAQ-CS were > 0.7, the values of GFI, TLI, and CFI were > 0.8, and RMSEA values ranged from 0.048–0.199. The mean of the safety attitudes of 2157 health professionals was 4.00, indicating a good safety attitude, with a positive response rate (% of items that scored ≥4) of 51.1%. The stress recognition subscale had the lowest score, with a mean of 2.73 and a positive response rate of 17.8%. A multiple linear regression equation revealed that demographic factors like gender, age, and training participation significantly affected the scores (β_gender_ > 0.06, β_age_ < − 0.08, β_training_ < − 0.07, *p* < 0.05).

**Conclusions:**

The psychometric properties of SAQ-CS are good and stable. Health professionals rate teamwork climate, safety climate, perception of management, and work conditions in Liaoning province are perceived as good; however, the stress of the health professionals is poor. To improve safety attitudes, it is necessary to not only reduce the stress of health professionals, but also to pay more attention to men, older health professionals, and health professionals who have not participated in safety training.

## Background

Patient safety is defined as the absence of preventable harm to a patient during the process of health care and acceptable minimum reduction of risk of unnecessary harm associated with health care [1]. Over the years, patient safety has been the focus of healthcare organizations worldwide. In 1999, the Institute of Medicine reported that approximately 44,000 to 98,000 persons died from preventable medical errors globally [2]. In European countries, the incidence of adverse events during healthcare delivery was about 8–12% [3]. Moreover, it is generally estimated that around 50% of adverse events in health care are preventable [4]. In 2016 and 2017, the World Health Organization conducted global patient safety summits in England and Germany and summarized existing problems and interventions in many countries [5, 6].

Questionnaires can be used to reflect the safety attitudes of health professionals, a proxy to instantaneous snapshots of the safety cultures, reflecting the weak points and potential hazards in the medical system. This allows health professionals to identify and change the working methods to reduce the occurrence of adverse events. Moreover, results can also provide baseline data for future research on safety culture [7]. The Safety Attitudes Questionnaire (SAQ) is a refinement of the Intensive Care Unit Management Attitudes Questionnaire [8, 9]. According to the recommendations of several studies, the SAQ has been adapted to several versions to suit different environments and populations [10–16]. Among different versions of the SAQ, the contents of the same item are similar, however, there are a few modifications done to best fit different environments. For example, a question has been modified from “In this ICU, it is difficult to discuss mistakes”, to “In the ORs here, it is difficult to discuss mistakes”, reflecting the proper work environment. The SAQ has been widely used in empirical studies in several Western countries, suggesting that safety attitudes of health professionals needed to be improved [17–21]. In Asian countries, such as mainland China, Korea, and Malaysia, the SAQ has also been used in empirical studies. In mainland China, Fanglei translated the original SAQ into Chinese. Responders were all nurses (*N* = 211) working in 6 units [22]. Xia translated the SAQ Ambulatory version into Chinese. Responders from six hospitals were doctors and nurses (*N* = 843) working in 8 units [23]. Moreover, taking the SAQ applied in Lee’s study as a reference, Xiuming and colleagues translated the SAQ Short Form into the Chinese Simplified form, utilizing five hospitals in Beijing with 1663 doctors, nurses, and technicians in 8 units. Cumulatively, these studies validate good psychometric properties of the SAQ, but suggest that safety attitudes of health professionals need to be improved [24–26].

Current studies on the safety attitudes of health professionals have several limitations, including small sample sizes, geographic limitations, and the small numbers and types of departments in which health professionals worked. Therefore, these studies cannot be generalized to safety attitudes of all Chinese health professionals. The aims of this study are, therefore, to test the reliability and validity of the SAQ, to measure the scores of nine tertiary hospitals, to group the samples by demographic factors with demographic factor levels for each subgroup for comparison between subgroups, and finally to explore the effects of demographic factors on SAQ scores.

## Methods

### Sample and survey administration

This study used a stratified sampling method to randomly choose nine tertiary hospitals in the Liaoning province and randomly select ten departments, including outpatients, wards, and technical units in each hospital. In Xi’s study [26], the mean of the total scale was 69.72 and the standard deviation was 15.47, according to the equation

$$ N={\left(\frac{CV\ast {Z}_{\alpha /2}}{\varepsilon}\right)}^2, $$

when ε is 2.75%, *N* = 250. We therefore decided to randomly choose 30 staff members for each department, yielding 300 staff members per hospital, to provide a sufficient sample size.

Inclusion criteria were that respondents must have worked in the department for at least 1 month prior to taking the survey, in order to know about the safety culture there. The study ran from June to August 2017. Before the survey, we invited experts to train the researchers how to fill out the questionnaire, and formed an instruction about filling out the SAQ. When the researchers arrived at the selected hospitals, they trained the head nurses from the selected departments to fill out questionnaires and distributed instructions, answer sheets, questionnaires and 2B pencils. Before morning shift or weekly meeting in every department, when nearly all the staffs were in the office, the head nurse distributed questionnaires and trained the staffs to fill out the questionnaire, then the dean and the head nurse supervised the staffs to fill out the questionnaires, at last the head nurses collected the questionnaires and returned them to the researchers. Two weeks were allowed to complete the questionnaires, the head nurses reminded the respondents to complete the surveys once a week. Retrieved questionnaires were scanned by an automatic optical checking machine to avoid errors. We used HPXScanManager software to export the data into an Excel file. Prior to filling out the survey, informed consent was obtained from each health professional and they participated voluntarily and anonymously. All answers were kept confidential and had no impact on the respondents’ work.

### Survey instrument

The SAQ was derived from the Intensive Care Unit Management Attitudes Questionnaire (ICUMAQ) and Flight Management Attitudes Questionnaire (FMAQ) [4, 9, 27, 28]. Previous studies showed a strong correlation between favorable SAQ scores and positive patient outcomes. Positive SAQ scores were associated with fewer medical errors, less ventilator-associated pneumonia, less catheter-related blood stream infections, and reduced length of hospital stay [29, 30]. Therefore, a higher SAQ score represented a better safety culture. The current study used the SAQ Chinese Simplified version adapted by Xi with an addition of two items that are not in subscales, from the version adapted by Lee. The two items not classified in subscales are “Administrators encourage the reporting of medical adverse events in this clinical area.” and “Managers prioritize safety training programs in this clinical area” [24, 26]. Table 1 provides the subscale titles with examples and number of items in each subscale for the SAQ. At the beginning of the questionnaire, we added demographic questions, including sex, age, highest degree received, and occupational position, function, technical title, years in the unit, years in the hospital, weekly work time, and participation in patient safety training, as these characteristics are likely to affect health professionals’ safety attitudes.

**Table 1:** Subscales and items in the SAQ

Subscale	Number of Items	Example
Teamwork Climate	6	Nurse input is well received in this clinical area.
Safety Climate	7	I would feel safe being treated here as a patient.
Job Satisfaction	5	I like my job.
Stress Recognition	4	When my workload becomes excessive, my performance is impaired.
Perception of Management	10	Management supports my daily efforts: Unit Mgt
		Management supports my daily efforts: Hosp Mgt
Work Conditions	4	The levels of staffing in this clinical area are sufficient to handle the number of patients.
No Subscale	7	My suggestions about safety would be acted upon if I expressed them to management.

### Statistical analysis

All items in the current study were assessed by using a five-point Likert scale: 1 = strongly disagree, 2 = disagree, 3 = neutral, 4 = agree and 5 = strongly agree. Items 2, 11, 20, 21, 22, 23, and 36 were reverse scored. In order to easily interpret the results, the Likert score was converted to a percentile score, with a Likert score of 1 corresponding to a percentile score of 0, recorded as 1 → 0, following 2 → 25, 3 → 50, 4 → 75 and 5 → 100. A score of less than or equal to 50 was deemed as needing improvement and a score of greater than or equal to 75 was deemed as a positive safety attitude. The scores of all the items in one subscale were summed, then divided by the number of items in that subscale to obtain the score of that subscale, ranging from 0 to 100. The percentages of the positive responses for each item were calculated, and percentages greater than or equal to 75% were considered to reflect a positive attitude [24, 26].

To test the reliability of the questionnaire, we calculated Cronbach’s alpha for each subscale, with a score over 0.70 suggesting good reliability. Confirmatory Factor Analysis (CFA) was applied to test the validity of the SAQ. Goodness-of-fit indices, namely Root Mean Square Error of Approximation (RMSEA), Comparative Fit Indices (CFI), Tucker-Lewis Index (TLI), and Goodness-of-Fit Index (GFI) were computed to demonstrate whether the entire model fit well. We considered a good model fit when values were as follows: RMSEA ≤0.08; CFI ≥ 0.90; TLI ≥ 0.90; and, GFI ≥ 0.90 [31, 32].

Percentages, means, and standard deviations were used to describe respondents’ characteristics and scale scores. A two-sample t-test was applied to compare the means between two groups and an ANOVA was applied to compare the means in several groups. Multiple linear regression analysis was applied to identify the demographic factors that could affect scores and the directions of the functions of these factors. Missing values were replaced by medians of items and then entered into the analysis [33]. Statistical significance was defined by *p* ≤ 0.05. CFA was performed using SPSS AMOS, version 17.0 (IBM, Armonk, New York, USA) and other analyses were performed with IBM SPSS, version 16.0 (IBM, Armonk, New York, USA).

## Results

### Response rate

A total of 2584 questionnaires were distributed; each unit entering the survey received 30 questionnaires and 10 units in each hospital participated in this survey. As one of the hospitals was an oncology hospital with no pediatrics unit, 9 units in this hospital participated in the survey. In some hospitals, there were less than thirty staff members in some units, in which case the number of the questionnaires administered equaled to the number of the staff in these units. In total, 2190 questionnaires were retrieved and considered valid, for a valid response rate of 84.75%.

### Reliability and validity of the scales

Cronbach’s alpha values ranged from acceptable to excellent for teamwork climate (α = 0.784), safety climate (α = 0.769), job satisfaction (α = 0.871), stress recognition (α = 0.876), perception of management (α = 0.918), work conditions (α = 0.751) and the total scale (α = 0.938).

The validity indices are shown in Table 2. The lowest value for GFI, TLI, and CFI was 0.880 and the highest value for RMSEA was 0.199.

**Table 2:** Goodness-of-fit indices for each subscale and the total scale

Subscale	GFI	TLI	CFI	RMSEA
TW	0.989	0.969	0.982	0.057
SC	0.979	0.941	0.961	0.069
JS	0.997	0.995	0.997	0.035
SR	0.959	0.887	0.962	0.199
PM	0.886	0.880	0.920	0.130
WC	0.993	0.963	0.988	0.083
Total	0.906	0.924	0.931	0.048

TW means teamwork climate; SC means safety climate; JS means job satisfaction; SR means stress recognition; PM means perception of management; WC means work conditions; Total means total scale

### Scale scores

Item and subscale scores are shown in Table 3. The means of all items were greater than 4, with the percentages of positive responses higher than 75% in the job satisfaction and perception of management subscales, thereby qualifying these two subscales to be considered as eliciting favorable safety attitudes. For the stress recognition subscale, the scores of all the items were lower than 3, therefore this subscale was considered as eliciting an inferior safety attitude.

**Table 3:** Means, standard deviations, and percentage of positive response to items

NO	Item	Mean	SD	PPR (%)
Total Scale		4.02	0.50	51.1
Teamwork Climate		4.20	0.61	71.0
1	Nurse input is well received in this clinical area.	4.09	0.89	78.4
2	In this clinical area, it is difficult to speak up if I perceive a problem with patient care.	3.90	1.13	71.8
3	Disagreements in this clinical area are resolved appropriately (i.e., not who is right, but what is best for the patient).	4.27	0.80	86.0
4	I have the support I need from other personnel to care for patients.	4.18	0.86	82.6
5	It is easy for personnel here to ask questions when there is something that they do not understand.	4.34	0.75	89.1
6	The physicians and nurses here work together as a well-coordinated team.	4.41	0.77	88.8
Safety Climate		4.08	0.58	61.4
7	I would feel safe being treated here as a patient.	4.37	0.76	88.8
8	Medical errors are handled appropriately in this clinical area.	4.31	0.75	87.8
9	I know the proper channels to direct questions regarding patient safety in this clinical area.	4.00	0.90	75.3
10	I receive appropriate feedback about my performance.	4.05	0.88	80.2
11	In this clinical area, it is difficult to discuss errors.	3.68	1.24	63.8
12	I am encouraged by my colleagues to report any patient safety concerns I may have.	3.90	0.92	70.9
13	The culture in this clinical area makes it easy to learn from the errors of others.	4.21	0.78	85.4
Job Satisfaction		4.25	0.67	74.0
15	I like my job.	4.12	0.90	78.5
16	Working here is like being part of a large family.	4.33	0.78	88.0
17	This is a good place to work.	4.30	0.81	86.2
18	I am proud to work in this clinical area.	4.28	0.79	85.0
19	Morale in this clinical area is high.	4.21	0.84	83.2
Stress Recognition		2.79	1.07	20.5
20	When my workload becomes excessive, my performance is impaired.	2.79	1.28	30.8
21	I am less effective at work when fatigued.	2.70	1.24	27.4
22	I am more likely to make errors in tense or hostile situations.	2.85	1.23	32.1
23	Fatigue impairs my performance during emergency situations (e.g. emergency resuscitation, seizure).	2.82	1.26	33.1
Perception of Management		4.20	0.61	71.7
24.1	Management supports my daily efforts: Unit Mgt	4.24	0.80	85.7
24.2	Management supports my daily efforts: Hosp Mgt	4.12	0.85	79.4
25.1	Management doesn’t knowingly compromise pt. safety: Unit Mgt	4.34	0.77	89.1
25.2	Management doesn’t knowingly compromise pt. safety: Hosp Mgt	4.30	0.78	87.5
26.1	Management is doing a good job: Unit Mgt	4.32	0.77	87.5
26.2	Management is doing a good job: Hosp Mgt	4.22	0.82	83.6
27.1	Problem personnel are dealt with constructively by our: Unit Mgt	4.16	0.81	82.8
27.2	Problem personnel are dealt with constructively by our: Hosp Mgt	4.09	0.84	80.0
28.1	I get adequate, timely info about events that might affect my work, from: Unit Mgt	4.15	0.81	82.0
28.2	I get adequate, timely info about events that might affect my work, from: Hosp Mgt	4.07	0.83	78.4
Work Conditions		4.04	0.66	62.4
29	The levels of staffing in this clinical area are sufficient to handle the number of patients.	3.65	1.12	61.6
30	This hospital does a good job of training new personnel.	4.20	0.81	83.7
31	All the necessary information for diagnostic and therapeutic decisions is routinely available to me.	4.09	0.79	79.0
32	Trainees in my discipline are adequately supervised.	4.22	0.74	86.5
Items not in subscales				
14	My suggestions about safety would be acted upon if I expressed them to management.	3.95	0.91	71.9
33	I experience good collaboration with nurses in this clinical area.	4.34	0.73	89.5
34	I experience good collaboration with staff physicians in this clinical area.	4.34	0.72	89.0
35	I experience good collaboration with pharmacists in this clinical area.	4.15	0.86	80.5
36	Communication breakdowns that lead to delays in delivery of care are common.	3.71	1.30	63.4
37	Administrators encourage the reporting of medical adverse events in this clinical area.	3.80	1.01	64.1
38	Managers prioritize safety training programs in this clinical areas.	4.24	0.79	83.4

PPR means percentage of positive response. Items 2, 11, 20, 21, 22, 23, 36 are reverse scored. Items not classified in subscales (37 & 38) are from Lee’s study [24]

### Respondent characteristics and scores

Respondents’ demographic characteristics and scores are shown in Table 4. There were significant differences in the scores of the stress recognition subscale by employment factor. Similarly, there were significant differences in the scores of the perception of management and work conditions subscales by years in the unit. There were also significant differences in the scores of the work condition subscale by weekly work time. There were many significant differences between the subsets of each factor.

**Table 4:** Characteristics and scores of the respondents

Characteristics	N (%)	TW	SC	JC	SR	PM	WC	TOTAL
Sex								
male	566 (25.84%)	4.14 (0.61)	4.03 (0.57)	4.23 (0.65)	2.62 (1.06)	4.14 (0.65)	4.04 (0.67)	3.96 (0.50)
female	1624 (74.16%)	4.22 (0.61)	4.09 (0.59)	4.26 (0.68)	2.85 (1.07)	4.22 (0.60)	4.04 (0.66)	4.04 (0.50)
*p*		< 0.05	< 0.05	0.457	< 0.05	< 0.05	0.882	< 0.05
Age								
< =30	863 (39.41%)	4.25 (0.59)	4.12 (0.58)	4.29 (0.66)	2.87 (1.07)	4.27 (0.60)	4.13 (0.65)	4.08 (0.50)
31–50	1182 (53.97%)	4.19 (0.61)	4.06 (0.59)	4.23 (0.68)	2.73 (1.08)	4.17 (0.62)	4.00 (0.67)	4.00 (0.50)
> =51	145 (6.62%)	3.96 (0.65)	3.93 (0.59)	4.14 (0.63)	2.79 (0.96)	4.03 (0.62)	3.88 (0.61)	3.87 (0.52)
*p*		< 0.05	< 0.05	< 0.05	< 0.05	< 0.05	< 0.05	< 0.05
Degree								
below bachelor	363 (16.70%)	4.08 (0.62)	3.97 (0.57)	4.16 (0.66)	2.81 (1.08)	4.13 (0.59)	3.95 (0.64)	3.94 (0.49)
bachelor	1113 (51.20%)	4.21 (0.60)	4.09 (0.59)	4.26 (0.70)	2.90 (1.07)	4.21 (0.62)	4.05 (0.66)	4.04 (0.51)
master	559 (25.71%)	4.25 (0.62)	4.11 (0.59)	4.29 (0.63)	2.64 (1.05)	4.22 (0.61)	4.08 (0.66)	4.04 (0.50)
Dr./PhD	139 (6.39%)	4.23 (0.57)	4.09 (0.54)	4.21 (0.61)	2.40 (1.03)	4.23 (0.67)	4.01 (0.72)	3.98 (0.46)
*p*		< 0.05	< 0.05	< 0.05	< 0.05	0.118	< 0.05	< 0.05
Occupation								
physician	765 (35.11%)	4.29 (0.58)	4.14 (0.58)	4.29 (0.64)	2.73 (1.12)	4.25 (0.62)	4.10 (0.64)	4.06 (0.49)
pharmacist	204 (9.36%)	3.99 (0.63)	3.91 (0.56)	4.17 (0.70)	2.58 (0.95)	4.08 (0.60)	3.85 (0.73)	3.87 (0.49)
nurse	883 (40.52%)	4.21 (0.59)	4.09 (0.59)	4.23 (0.68)	2.95 (1.08)	4.22 (0.60)	4.07 (0.64)	4.04 (0.51)
technician	291 (13.36%)	4.10 (0.64)	3.97 (0.57)	4.25 (0.68)	2.63 (0.96)	4.12 (0.65)	3.94 (0.70)	3.93 (0.51)
other	36 (1.65%)	4.31 (0.49)	4.19 (0.48)	4.26 (0.55)	2.65 (1.00)	4.31 (0.52)	4.08 (0.61)	4.07 (0.42)
*p*		< 0.05	< 0.05	0.151	< 0.05	< 0.05	< 0.05	< 0.05
Function								
dean	48 (2.22%)	4.25 (0.58)	4.06 (0.54)	4.33 (0.60)	3.10 (1.14)	4.28 (0.53)	4.02 (0.58)	4.07 (0.45)
head nurse	61 (2.82%)	4.21 (0.65)	4.08 (0.53)	4.30 (0.68)	2.99 (1.05)	4.23 (0.56)	4.13 (0.58)	4.08 (0.50)
staff	1943 (89.79%)	4.21 (0.60)	4.08 (0.59)	4.26 (0.67)	2.80 (1.08)	4.20 (0.61)	4.04 (0.67)	4.02 (0.50)
other	112 (5.17%)	4.13 (0.67)	4.04 (0.62)	4.12 (0.67)	2.48 (0.90)	4.19 (0.64)	4.02 (0.64)	3.94 (0.53)
*p*		0.603	0.901	0.127	< 0.05	0.883	0.718	0.243
Technical title								
none	222 (10.26%)	4.18 (0.64)	4.07 (0.57)	4.22 (0.68)	2.92 (1.11)	4.25 (0.62)	4.12 (0.66)	4.04 (0.52)
junior	902 (41.70%)	4.25 (0.58)	4.13 (0.57)	4.31 (0.65)	2.80 (1.06)	4.25 (0.60)	4.10 (0.66)	4.07 (0.49)
intermediate	720 (33.29%)	4.13 (0.63)	4.01 (0.62)	4.17 (0.70)	2.74 (1.07)	4.13 (0.62)	3.96 (0.66)	3.95 (0.51)
sub-senior	216 (9.99%)	4.25 (0.54)	4.08 (0.53)	4.26 (0.63)	2.75 (1.10)	4.20 (0.60)	4.03 (0.67)	4.02 (0.49)
senior	103 (4.76%)	4.18 (0.62)	4.05 (0.57)	4.30 (0.66)	2.89 (1.06)	4.17 (0.63)	4.02 (0.64)	4.02 (0.51)
*p*		< 0.05	< 0.05	< 0.05	0.198	< 0.05	< 0.05	< 0.05
Years in unit								
< 3y	586 (26.79%)	4.22 (0.63)	4.09 (0.58)	4.29 (0.66)	2.73 (1.05)	4.26 (0.61)	4.12 (0.66)	4.05 (0.50)
3-12y	1149 (52.54%)	4.21 (0.59)	4.08 (0.58)	4.24 (0.68)	2.79 (1.08)	4.19 (0.61)	4.03 (0.66)	4.02 (0.50)
> 12y	452 (20.67%)	4.15 (0.62)	4.03 (0.59)	4.23 (0.67)	2.86 (1.08)	4.16 (0.62)	3.97 (0.66)	3.99 (0.51)
*p*		0.159	0.194	0.247	0.146	< 0.05	< 0.05	0.156
Years at hospital								
< 3y	446 (20.68%)	4.25 (0.65)	4.13 (0.60)	4.33 (0.68)	2.72 (1.01)	4.31 (0.62)	4.16 (0.67)	4.08 (0.51)
3-12y	1144 (53.04%)	4.22 (0.57)	4.08 (0.57)	4.24 (0.65)	2.81 (1.09)	4.18 (0.60)	4.03 (0.66)	4.02 (0.49)
> 12y	567 (26.29%)	4.12 (0.64)	4.01 (0.60)	4.20 (0.70)	2.83 (1.07)	4.14 (0.62)	3.95 (0.65)	3.97 (0.52)
*p*		< 0.05	< 0.05	< 0.05	0.224	< 0.05	< 0.05	< 0.05
Weekly work time								
< =40 h	270 (12.48%)	4.18 (0.61)	4.06 (0.55)	4.26 (0.68)	2.82 (0.95)	4.25 (0.59)	4.18 (0.64)	4.04 (0.49)
40-48 h	1479 (68.34%)	4.19 (0.61)	4.07 (0.59)	4.26 (0.67)	2.81 (1.09)	4.21 (0.61)	4.03 (0.67)	4.02 (0.51)
> =56 h	415 (19.18%)	4.23 (0.58)	4.09 (0.57)	4.21 (0.66)	2.68 (1.11)	4.15 (0.64)	3.99 (0.65)	3.99 (0.49)
*p*		0.372	0.772	0.382	0.069	0.095	< 0.05	0.373
Participation in training								
yes	1465 (67.64%)	4.27 (0.58)	4.14 (0.57)	4.30 (0.65)	2.86 (1.08)	4.25 (0.59)	4.11 (0.63)	4.08 (0.49)
no	701 (32.36%)	4.05 (0.64)	3.95 (0.59)	4.13 (0.70)	2.65 (1.05)	4.09 (0.65)	3.89 (0.70)	3.89 (0.51)
*p*		< 0.05	< 0.05	< 0.05	< 0.05	< 0.05	< 0.05	< 0.05

TW means teamwork climate, SC means safety climate, JS means job satisfaction, SR means stress recognition, PM means perception of management, WC means work conditions, TOTAL means total scale. h means hour, m means month, y means year

### Regression analysis

Multiple linear regression was conducted to analyze the effect of demographic characteristics on scores (see Table 5 for results). Women generally scored higher than men, the longer time in the unit, the higher the score, and higher age was related to a lower score. General staff scored higher than head nurses and deans, while the more years worked in the hospital, the lower the score. Similarly, more hours per week worked, the lower the score. Persons who attended training scored higher than those who have not, while those with a more advanced degree scored lower than those with lower degree in subscale teamwork climate and stress recognition, but in other scales, people with the more advanced degree scored higher.

**Table 5:** Multiple linear regression analysis of the effects of demographic characteristics on safety attitude scores

Subscale	Variable									
	Sex	Age	Degree	Occupation	Function	Technical title	Years in unit	Years in hospital	Weekly work time	Participation in training
TW	√	√	√	√	√	–	–	–	–	√
Beta	0.063	−0.107	− 0.097	0.021	−0.040	–	–	–	–	− 0.183
*P*	< 0.05	< 0.05	< 0.05	0.351	0.082	–	–	–	–	< 0.05
SC	√	–	√	√	√	–	–	√	–	√
Beta	0.065	–	0.071	0.017	−0.027	–	–	− 0.085	–	− 0.162
*P*	< 0.05	–	< 0.05	0.453	0.243	–	–	< 0.05	–	< 0.05
JS	–	–	–	√	√	–	–	√	–	√
Beta	–	–	–	0.017	−0.065	–	–	− 0.085	–	− 0.118
*P*	–	–	–	0.465	< 0.05	–	–	< 0.05	–	< 0.05
SR	√	√	√	√	√	–	√	–	–	√
Beta	0.068	−0.103	− 0.079	− 0.007	− 0.058	–	0.080	–	–	− 0.079
*P*	< 0.05	< 0.05	< 0.05	0.739	< 0.05	–	< 0.05	–	–	< 0.05
PM	√	√	√	√	√	–	–	–	–	√
Beta	0.065	−0.099	0.062	0.022	−0.025	–	–	–	–	− 0.135
*P*	< 0.05	< 0.05	< 0.05	0.328	0.268	–	–	–	–	< 0.05
WC	–	√	√	√	√	–	–	√	√	√
Beta	–	−0.085	0.059	−0.004	− 0.030	–	–	− 0.067	− 0.073	− 0.169
*P*	–	< 0.05	< 0.05	0.843	0.189	–	–	< 0.05	< 0.05	< 0.05
Total	√	√	√	√	–	–	–	–	–	√
Beta	0.069	−0.117	0.057	0.015	−0.051	–	–	–	–	− 0.178
*P*	< 0.05	< 0.05	< 0.05	0.510	< 0.05	–	–	–	–	< 0.05

√ means this variable was entered in the regression equation. Beta means standard Beta. TW means teamwork climate, SC means safety climate, JS means job satisfaction, SR means stress recognition, PM means perception of management, WC means work conditions, Total means total scale

## Discussion

Our study showed that the response rate was 84.75%, which was higher than that in Lee’s and Sexton’s studies (69.4 and 67.0%, respectively), this indicated the results could reflect the respondents’ safety attitudes more accurately [24, 34]. Moreover, the psychometric properties of safety attitudes questionnaire was good, which indicated the results were reliable. The Cronbach’s alpha values ranged from 0.751 to 0.938, the results were similar to those in Xi’s and Lee’s studies (scopes of Cronbach’s alpha values were 0.785–0.945 and 0.785–0.912, respectively), indicating the reliability of SAQ was stable [24, 26]. Furthermore, the lowest value of GFI, CFI and TLI was 0.880 and the highest RMSEA value was 0.199, indicating a good model fit in this study. In Xi’s study, the values of GFI and CFI were 0.948 and 0.963, and the values of the validity indices ranged from 0.97–1.00 in Lee’s study [24, 26, 34]. Cumulatively, these studies have shown a stable validity of the SAQ, rendering it is suitable for measuring the health professionals’ safety attitudes in mainland China and Taiwan.

Firstly, the scores of the teamwork climate and safety climate subscales were good; only a few item scores were low. The scores of items 2, 9, 11, and 12 were lower than those of other items, different from the results in Zimmermann’s study, in which items 1, 2, 8, 9, and 11 had higher means than those of other items [19]. The current study, therefore, suggests creating a non-punitive and open culture, establishing simple and expeditious channels, training health professionals to report adverse events, and encouraging health professionals to discuss adverse events and report them in a timely manner. Conversely, this subscale obtained low scores in Raftopouos’ study, implying more attention should be paid to infrastructure and leadership attitudes on handling of errors and learning from adverse events [16].

Secondly, the scores of the job satisfaction subscale items were higher than those in other subscales, which was similar to the results in the studies of Nordenhagg, Patterson, Raftopoulos, Kristensen and Bondevik [11–13, 34, 35]. The current study suggests that when hospitals created a climate that allows health professionals to feel family warmth when they worked, a considerable number of health professionals stated loving their work and having high work morale and acknowledged that this would be beneficial to patient safety.

Contrary to previous research, the perception of management subscale items in the current study scored higher than those in other subscales. The international baseline data showed that the means of the items ranged from 38.3 to 55.3 [34]. This subscale score was low in the studies of Nguyen, Relihan and Sexton, scores were 49.4, 48.0 and 38.3, respectively [17, 34, 35]. Indeed, management in the Liaoning province hospitals was good, taking the events that affecting health professionals’ work seriously, particularly errors affecting patient safety, which can improve health professionals’ safety attitudes. It should be noted that serious hierarchical structure can prevent unit staffs from speaking out or discussing safety problems to the management, so it can affect safety attitude [17]. Focusing on prevention, monitoring, learning and improvement on management approach to patient safety, not on blaming and punishing, can benefit patient safety [18]. Moreover, leadership walkrounds applied in studies showed enough power in improving perception of management [35].

It should be noted that the stress recognition subscale obtained the lowest scores of all the subscales, which were similar to those in the studies of Raftopoulos, Xi and Haerkens [10, 26, 36]. However, in contrast previous results, the international benchmark data showed that the means in this subscale for OR-UK was only a score of 54.7, but the means of other samples obtained scores higher than 64 [34]. Moreover, studies of Nguyen and Relihan showed that this subscale obtained mean scores higher than 74 [17, 35]. Results from the current study implies that, when compared to other countries, the safety attitudes of the health professionals in the Liaoning province may be affected by stress more easily, which could affect patient safety. One possible explanation for this difference may be that with China’s rapid economic development, patients’ expectations for a better healthcare service exceed the current capabilities. Moreover, the triage system is not good enough and the existing setup of most primary care clinics cannot meet the patients’ expectations. Therefore, most patients choose tertiary hospitals, overloading the doctor-patient ratio, leading to an increase in health professionals’ workloads. Moreover, subsequent factors including occupational stress, fatigue, burnout, job dissatisfaction and sleep deprivation will jeopardize patient safety [26, 37–40]. Therefore, adequate staffing levels are necessary to decrease stress [34]. Moreover, there are inherently risks in medical service, the development of medical services often lags behind the development of diseases, if patients were harmed in adverse events, and the relationship between patients and doctors will be tense, inevitably increasing health professionals’ work pressure, and making health professionals perform worse than usual, ultimately, these will affect patient safety.

The results showed most items in the work conditions subscale obtained favorable scores, but item 29 obtained a lower score, which was similar to those in Xi’s study [26]. Conversely, the results in Samsuri’s study showed that this subscale score was 54.8, lower than other subscales [16]. Studies implied that training and supervising newcomers and maintaining diagnostic and therapeutic information would be beneficial to patient safety. Moreover, to build a better working condition, we should keep adequate staffing, training and supervising new comers, availability of information for therapeutic decision [16], [41]. Subscale means and standard deviations in other empirical researches that were compared with this study were shown in Table 6.

**Table 6:** Subscale means and standard deviations in empirical researches

No.	Researcher	Reference	Teamwork Climate	Safety Climate	Job Satisfaction	Stress Recognition	Perception of Management	Working Condition	Overall
1	Raftopoulos V	[10]	74.1 (14.4)	73.1 (13.7)	82.4 (12.9)	30.6 (14.4)	54.6 (27.0)	67.0 (20.4)	–
2	Nordenhagg A	[14]	83.20 (18.26)	80.43 (15.61)	82.62 (20.17)	72.15 (22.73)	70.25 (21.64)	73.23 (20.83)	–
3	Raftopoulos V	[16]	57.95 (21.17)	55.82 (18.97)	66.20 (22.64)	50.64 (18.77)	52.14 (19.69)	55.03 (20.83)	–
4	Nguyen G	[17]	66.4 (16.3)	65.1 (14.9)	70.6 (22.1)	75.8 (22.7)	49.4 (24.0)	51.6 (23.7)	–
5	Buljacsamardzic M	[18]	70.1 (13.1)	64.5 (12.4)	69.9 (14.6)	52.7 (18.9)	55.1 (15.5)	56.1 (15.8)	–
6	Kristensen S	[20]	77.2 (15.7)	70.3 (16.8)	76.2 (17.7)	68.1 (22.7)	66.8 (20.6)	73.8 (22.0)	–
7	Lee W	[24]	48.9 (11.8)	37.2 (11.4)	42.1 (12.9)	–	45.2 (13.9)	31.8 (13.5)	–
8	Cui Y	[26]	74.87 (18.23)	73.82 (17.51)	72.43 (22.50)	44.53 (28.70)	69.64 (19.68)	68.59 (20.09)	69.72 (15.47)
9	Sexton JB	[34]	64.3 (16.6)	60.5 (16.0)	59.6 (20.5)	74.4 (20.2)	38.3 (18.7)	49.2 (19.5)	–
10	Relihan EC	[35]	73.7 (14.9)	71.0 (15.8)	67.9 (19.4)	74.7 (17.1)	48.0 (19.2)	58.2 (21.9)	–
11	Poley MJ	[37]	69.0 (12.1)	69.4 (14.2)	65.6 (13.9)	52.2 (16.4)	55.4 (12.7)	54.4 (11.8)	–
12	Haerkens MH	[36]	3.60 (0.58)	3.47 (0.57)	3.65 (0.65)	2.99 (0.73)	3.01 (0.66)	3.13 (0.56)	–
13	Samsuri SE	[40]	67.6 (14.5)	66.8 (14.9)	67.3 (19.4)	73.0 (20.4)	62.2 (14.0)	54.8 (17.4)	65.6 (11.0)

The scores of the items not in subscales showed that strengthening the communication of health professionals, encouraging health professionals to report adverse events, and making safety training a priority would be beneficial to patient safety. Items 38 obtained a high score, suggesting that safety training received enough attention. Item 36 obtained a low score, but was reverse worded and may have been misunderstood. We therefore suggest that future studies should positively word this item to decrease respondents’ misunderstanding. The current study obtained results of the scores of items not in subscales similar to those previously reported by Xi and Lee [24, 26], except for item 37, which was rated low, contrary to Lee’s study. These results suggest that managers should encourage reporting adverse events. If adverse events are to be resolved, the first step is to report them, however, when reporting adverse events, the reputation of individuals and hospitals may be affected. Moreover, if the adverse events are discovered by patients and their families, health professionals may experience unnecessary disputes and lawsuits. Therefore, managers should improve the mechanism of medical disputes and strengthen the sharing mechanism of medical risks to allow health professionals to safely and promptly report adverse events [24, 26].

This study explored the impacts of demographic factors on safety attitudes. Sex, age, degree, occupational function, technical title, years in hospital, and participation in training all significantly affected safety attitudes. Further analyses were conducted to determine the direction of the influences of these factors on safety attitudes. Females achieved higher scores than males in most subscales, possibly due to the fact that females are more careful when they do things. Our results was similar to those in Kristensen’s study [20]. Additionally, scores tended to decrease with increasing age, perhaps because older individuals had more work experience and therefore experienced more patient risks. Moreover, the results in Raftopoulos’ study showed younger nurses felt more powerful to cope with stressors [10]. The higher the degree earned, the lower the teamwork climate and stress recognition scores, potentially because highly educated health professionals have a wide ranger of ideas and think more about the problems, which may lead to more pressure, thereby affecting teamwork climate. General staff generally obtained lower scores in the job satisfaction and stress recognition subscales as well as on the total scale when compared to deans and head nurses, plausibly because general staffs have longer contacting time with patients, leading to the potential of more medical risks. Our results were similar to those in Relihan’s study, it implies the higher scores obtained by the head nurses may be explained by a sense of unit ownership and responsibility [35]. Additionally, health professionals with more years in the unit scored higher on stress recognition, which, due to reverse scoring, suggests that being familiar with units may decrease stress. However, the more years worked in the hospital the lower the safety attitudes score, possibly because of an increased knowledge about the defects in the work climate and conditions. Similarly, as weekly work time increased, health professionals had lower scores, suggesting that extending the time worked during the week may not improve patient safety. In all subscales, health professionals who participated in patient safety training obtained higher scores than those who did not participate in training, which demonstrated that training is an effective method to improve patient safety. This study suggests that increased attention should be paid to men, older individuals, those with an advanced degree, those with general staff positions, and staff who have worked in hospitals for a long time. Moreover, managers should ensure proper promotions, arrange workload reasonably, especially arrange multidisciplinary team to share responsibility [18], and promote safety training. These methods will improve patient safety.

### Strengthens and limitations

First, the selected hospitals in this study are representatives of the three-level hospitals in Liaoning Province, selected departments (including wards, outpatients, medical and technical departments) and respondents (including doctors, nurses and technicians) can represent the safety attitudes of health professionals in Liaoning Province. Second, the response rate is high, so the results can reflect the safety attitudes of the respondents accurately. Third, this study provides the influences of demography factors on safety attitudes, and provides reference for future intervention studies.

In spite of the many strengths, the current study also has some limitations. For example, there was no continuous assessment of safety attitudes and adverse event reports over time. In addition, there was no intervention to improve patient safety in this study; therefore, we suggest that a prospective study should include interventional methods to improve patient safety.

## Conclusions

The reliability and validity of the SAQ revised by Xi are good and this SAQ is suitable for measuring health professionals’ safety attitudes in the Liaoning province. The safety attitudes of health professionals in tertiary hospitals in the Liaoning province are good, these health professionals are satisfied with their work, and good management has promoted patient safety, but the health professionals’ stress was serious. This study provides baseline data for further research. Age, degree earned, occupational function, years in the hospital, weekly work time, and participation in training have negative impacts on safety attitudes. It is suggested that in future research it is necessary to strengthen the dynamic observation of safety attitudes and adverse events. In practice, it is necessary to cultivate an open and non-punitive culture to encourage and train health professionals to report adverse events. Moreover, it is necessary to establish a scientific and reasonable hierarchical diagnosis and treatment system, arrange health professionals’ workload reasonably, increase the number of health professionals reasonably, and reduce health professionals’ stress.

## Data Availability

The datasets used and/or analysed during the current study are available from the corresponding author on reasonable request.

## Abbreviations

CFA: Confirmatory Factor Analysis.
CFI: Comparative Fit Index
CV: Coefficient of variation
FMAQ: Flight management attitudes questionnaire
GFI: Goodness-of-Fit
ICUMAQ: Intensive care unit management attitudes questionnaire
RMSEA: Root Mean Square Error of Approximation
SAQ: Safety Attitudes Questionnaire
TLI: Tucker-Lewis Index
WHO: World Health Organization

## References

[1] Patient Safety. World Health Organization. https://www.who.int/patientsafety/en/. Accessed 30 Jan 2016.

[2] Kohn LT, Corrigan JM, Donaldson MS (2000). To Err is Human. Science.

[3] Muiño Miguez A., Jiménez Muñoz A.B., Pinilla Llorente B., Durán García E., Rodríguez Pérez M.P. (2007). Seguridad del paciente y calidad asistencial. Revista Clínica Española.

[4] Zwart DLM, Langelaan M, De Vooren RCV, Kuyvenhoven MM, Kalkman CJ, Verheij TJM, Wagner C (2011). Patient safety culture measurement in general practice. Clinimetric properties of ‘SCOPE’. BMC Fam Pract.

[5] Angela Y. Patient Safety 2030. NIHR Patient Safety Translational Research Centre at Imperial College London and Imperial College Healthcare NHS Trust. http://www.imperial.ac.uk/media/imperial-college/institute-of-global-health-innovation/centre-for-health-policy/Patient-Safety-2030-Report-VFinal.pdf. Accessed 30 Mar 2016.

[6] Margaret C. Best practices in Patient Safety. World Health Organization https://www.bundesgesundheitsministerium.de/fileadmin/Dateien/3_Downloads/P/Patientensicherheit/Best-Practice_Patient_Safety_Web_plusWHO.pdf. Accessed 1 May 2017.

[7] Guldenmund FW (2000). The nature of safety culture: a review of theory and research. Saf Sci.

[8] Sexton JB, Thomas EJ (2000). Helmreich RL etal. Error, stress, and teamwork in medicine and aviation: cross sectional surveys. BMJ.

[9] Thomas EJ, Sexton JB, Helmreich RL (2003). Discrepant attitudes about teamwork among critical care nurses and physicians. Crit Care Med.

[10] Raftopoulos V, Pavlakis A (2013). Safety climate in 5 intensive care units: a nationwide hospital survey using the Greek-Cypriot version of the safety attitudes questionnaire. J Crit Care.

[11] Goras C, Wallentin FY, Nilsson U (2013). Swedish translation and psychometric testing of the safety attitudes questionnaire (operating room version). BMC Health Serv Res.

[12] Deilkas E, Hofoss D (2008). Psychometric properties of the Norwegian version of the Safety Attitudes Questionnaire (SAQ), generic version (short form 2006). BMC Health Serv Res.

[13] Modak I, Sexton JB, Lux TR (2007). Measuring safety culture in the ambulatory setting: the safety attitudes questionnaire--ambulatory version. J Gen Intern Med.

[14] Nordenhagg A, Sexton JB, Kalvemarksporrong S (2010). Assessing Safety culture in pharmacies: the psychometric validation of the Safety attitudes questionnaire (SAQ) in a national sample of community pharmacies in Sweden. BMC Clin Pharmacol.

[15] Patterson PD, Huang D, Fairbanks RJ (2010). The emergency medical services safety attitudes questionnaire. Am J Med Qual.

[16] Raftopoulos V, Savva N, Papadopoulou M (2011). Safety culture in the maternity units: a census survey using the safety attitudes questionnaire. BMC Health Serv Res.

[17] Nguyen G, Gambashidze N, Ilyas SA (2015). Validation of the safety attitudes questionnaire (short form 2006) in Italian in hospitals in the northeast of Italy. BMC Health Serv Res.

[18] Buljacsamardzic M, Van Wijngaarden JD, Doorn CM (2016). Safety culture in long-term care: a cross-sectional analysis of the Safety attitudes questionnaire in nursing and residential homes in the Netherlands. BMJ Qual Saf.

[19] Zimmermann N, Kung K, Sereika SM (2013). Assessing the safety attitudes questionnaire (SAQ), German language version in Swiss university hospitals - a validation study. BMC Health Serv Res.

[20] Kristensen S, Sabroe S, Bartels P (2015). Adaption and validation of the Safety attitude questionnaire for the Danish hospital setting. Clin Epidemiol.

[21] Profit J, Etchegaray JM, Petersen LA (2012). The Safety attitudes questionnaire as a tool for benchmarking safety culture in the NICU. Arch Dis Child Fetal Neonatal Ed.

[22] Chen FL, Zhou L (2009). Patient safety culture assessment scale: design and establishment. Nurs J Shin PLA.

[23] Guo X, Zhou W (2010). Revision of Safety attitudes questionnaire and evaluation. Chin J Nurs Educ.

[24] Lee W, Wung H, Liao H (2010). Hospital Safety culture in Taiwan: a nationwide survey using chinese version safety attitude questionnaire. BMC Health Serv Res.

[25] Deng XX, Xi XM, Cui Y (2015). Evaluation of reliability and validity of Chinese version safety attitudes questionnaire using to assess patient safety culture. Med Soc.

[26] Cui Y, Xi XM, Zhang JS (2017). The safety attitudes questionnaire in Chinese: psychometric properties and benchmarking data of the safety culture in Beijing hospitals. BMC Health Serv Res.

[27] Vincent C, Tayloradams S, Stanhope N (1998). Framework for analysing risk and safety in clinical medicine. BMJ.

[28] Donabedian A (1988). The quality of care. How can it be assessed. JAMA.

[29] Colla J, Bracken AC, Kinney LM (2005). Measuring patient safety climate: a review of surveys. Qual Saf Health Care.

[30] Robb G, Seddon M. Measuring the safety culture in a hospital setting: a concept whose time has come?. The New Zealand Medical Journal. 2010;123:1314. 20581914

[31] Vandenberg RJ, Lance CE (2000). A review and synthesis of the measurement invariance literature: suggestions, practices, and recommendations for organizational research. Organ Res Methods.

[32] Browne MW, Cudeck R (1992). Alternative ways of assessing model fit. Sociol Methods Res.

[33] Huisman M (2000). Imputation of missing item responses : some simple techniques. Qual Quant.

[34] Bondevik GT, Hofoss D, Hansen EH, et al. The safety attitudes questionnaire –ambulatory version: psychometric properties of the Norwegian translated version for the primary care setting. BMC Health Services Research. 2014;14(1):139-9. 10.1186/1472-6963-14-139 PMC3994245 24678764

[35] Poley Marten J., van der Starre Cynthia, van den Bos Ada, van Dijk Monique, Tibboel Dick (2011). Patient safety culture in a Dutch pediatric surgical intensive care unit: An evaluation using the Safety Attitudes Questionnaire. Pediatric Critical Care Medicine.

[36] Relihan E., Glynn S., Daly D., Silke B., Ryder S. (2009). Measuring and benchmarking safety culture: application of the safety attitudes questionnaire to an acute medical admissions unit. Irish Journal of Medical Science.

[37] Kaya S, Barsbay S, Karabulut E, et al. The Turkish version of the safety attitudes questionnaire: psychometric properties and baseline data. Quality & Safety in Health Care. 2010;19(6):572-7. 10.1136/qshc.2008.032003 20671082

[38] Doctor AZ, Professor ME, Nurse RN, et al. Assessing safety culture in NICU: psychometric properties of the Italian version of Safety Attitude Questionnaire and result implications. J Evaluation in Clin Practice. 2016;22:275-82. 10.1111/jep.12472 26494199

[39] Haerkens MH, Van Leeuwen W, Sexton JB, et al. Validation of the Dutch language version of the Safety Attitudes Questionnaire (SAQ-NL). BMC Health Services Research. 2016;16(1):385. 10.1186/s12913-016-1648-3 PMC4986249 27528393

[40] Sexton JB, Helmreich RL, Neilands TB, et al. The Safety Attitudes Questionnaire: psychometric properties, benchmarking data, and emerging research. BMC Health Services Research. 2006;6(1):44-44. 10.1186/1472-6963-6-44 PMC1481614 16584553

[41] Samsuri Srima Elina, Pei Lin Lua, Fahrni Mathumalar Loganathan (2015). Safety culture perceptions of pharmacists in Malaysian hospitals and health clinics: a multicentre assessment using the Safety Attitudes Questionnaire. BMJ Open.

